# Association of *β*-casein gene polymorphism with milk composition traits of Egyptian Maghrebi camels (*Camelus dromedarius*)

**DOI:** 10.5194/aab-63-493-2020

**Published:** 2020-12-22

**Authors:** Amira M. Nowier, Sherif I. Ramadan

**Affiliations:** 1 Biotechnology Research Department, Animal Production Research Institute, Agriculture Research Center, Dokki, Egypt; 2 Animal Wealth Development Department, Faculty of Veterinary Medicine, Benha University, Toukh, Egypt

## Abstract

The objectives of this study were to detect the polymorphism of 2126${}_{A/G}$ SNP in the β-casein (CSN2) gene among Egyptian Maghrebi camels and to investigate the association of 2126${}_{A/G}$ SNP genotypes, parity, lactation stage, and temperature–humidity index (THI) with the milk composition traits of Maghrebi camels. Sixty-eight hair samples were collected from three different populations of Maghrebi camels for DNA extraction. Fat, protein, total solids, solids-not-fat, and lactose percentages were determined in Maghrebi camel milk using an automatic milk analyzer device. Three different genotypes – A/A, A/G, and G/G – were identified in the 5${}^{\prime }$ flanking region of β-casein gene by using PCR-RFLP method with the A/G genotype showing the highest frequency. Association among these three genotypes with milk composition traits suggests a positive effect of A/A genotype on acidity and protein percentage. Higher protein and acidity values were observed in the milk of individuals carrying the A/A genotype. The protein percentage of this study significantly increased from the first till the fourth parity and then decreased. Fat and total solid percentages were significantly higher in the late stage of lactation, while lactose showed a decreasing trend from the early till the late stages of lactation. Fat and protein percentages were highest in the low THI class. Our results encourage the utilization of Maghrebi camel milk for cheese and butter processing at the late lactation stages of the middle parities of their productive life. Moreover, the A/G SNP of the *CSN2* gene may be used as a DNA marker in selection programs for the improvement of camel milk composition. Further studies are needed in order to fully explore the variation in the chemical composition of camel milk due to the effect of CSN2 gene, parity, lactation stage, and THI factors.

## 1 Introduction

Camels are multipurpose animals used for meat and milk production, agricultural work and transportation, and for racing contests and tourism. In Egypt, there are different dromedary camel breeds: Maghrebi, used for milk and meat production; Somali and Sudani for racing; and Falahy for agricultural work (Wardeh et al., 1991; Ramadan et al., 2018; Nowier et al., 2020). In recent years, a growing demand for camel milk and processed milk products like pasteurized milk, flavored milk, fermented milk, milk tea, cheese, and butter has driven the commercialization of dairy camel farming (Ahmed and Kanwal, 2004; El-Agamy, 2009; Al-Saleh et al., 2011; Berhe et al., 2013). Products of camel milk are a good source of energy and nutrients, and they improve the limited shelf life of camel milk (Brezovečki et al., 2015). Therefore, intensive dairy camel farms are being established in the Gulf countries, Tunisia, Australia, Europe, and the USA to supply the local and international markets (Hammadi et al., 2010; Ayadi et al., 2013; Nagy and Juhasz, 2016; Faye, 2018).

Camel milk has many nutritional and therapeutic values, and it is more similar to human milk, because it contains a higher concentration of β-casein (65 %) and lower concentrations of κ-casein (3.5 %), αs1-casein (22 %), and αs2-casein (9.5 %) of total casein percentage than cow's milk, which might be the reason for its better digestibility and decreased frequency of allergies in infants (Berhe et al., 2017). The world's annual camel milk production has increased 4.6-fold over the last 50 years; in 1961 it was $0.63\times 10^{6}$ t; then it became $2.9\times 10^{6}$ t in 2013 (FAO, 2017). Due to the increase in camel milk production and processing, it is very important to monitor the chemical composition of camel milk and to study the various genetic and non-genetic factors that affect milk composition and processing properties (Nagy et al., 2017). Previous studies have shown that breed (Aljumaah et al., 2012), parity (Zeleke, 2007; Aljumaah et al., 2012), stage of lactation (Zeleke, 2007; Musaad et al., 2013), season (Zeleke, 2007; Ahmad et al., 2012), temperature–humidity index (THI; Abdel-Hameed, 2011; Ahmad et al., 2012), and feeding (Al-Saiady et al., 2012) are the major factors affecting the composition of dromedary camel milk. Recently, the THI has been broadly used all over the world as an indicator of the degree of stresses caused by weather conditions on livestock. THI allows for the combination of ambient temperature and relative humidity into one value, so comparison of environmental conditions can be made objectively (Abdel-Hameed, 2011; Ahmad et al., 2012; El-Tarabany and El-Tarabany, 2015).

The distribution of calcium and the stability of casein micelle are reported to be influenced by the different levels of β-casein phosphorylation, so β-casein is an important component of camel milk, and it plays a vital role in the nutritional and processing properties of camel milk and its products (Amigo et al., 2000). The β-casein (CSN2) gene coding for β-casein of dromedary camels consists of 9 exons and extends over 7819 nucleotides (Pauciullo et al., 2014). In ruminants, several studies have shown the association of β-casein gene polymorphism with economic important traits such as yield and composition of milk in cattle (Huang et al., 2012; Viale et al., 2017; Soyudal et al., 2018), in buffalo (Singh et al., 2007), in sheep (Corral et al., 2010), and in goats (Cosenza et al., 2007). Previously, Pauciullo et al. (2014) identified a transition g.2126${}_{A>G}$ in CSN2 promoter region of four Sudanese *Camelus dromedarius* populations. This 2126${}_{A/G}$ SNP is located three nucleotides downstream of the TATA box and might modify the binding affinity of RNA polymerase and change the gene expression (Lee et al., 2012). Conversely, although this gene is important in camels, there are no studies that investigate the possible association of this gene with milk composition traits. Therefore, the objectives of this study were to investigate the effect of genetic factors such as β-casein gene polymorphism and the effect of non-genetic factors such as parity, lactation stage, and THI on milk chemical composition traits in Maghrebi camels. To our knowledge, there is no available study that has evaluated the association between β-casein gene polymorphism with chemical composition of camel milk.

## 2 Materials and methods

### 2.1 Compliance with ethical standards

All procedures performed in studies involving animals were in accordance with the ethical standards of the institution or practice at which the studies were conducted (Committee of Animal Care and Welfare, Benha University, Egypt), with approval number BUFVTM 2019.

### 2.2 Experimental populations and management

Sixty-eight female Maghrebi camels (*Camelus dromedarius*) belonging to three different populations (farms) located in Mersa Matrouh governorate in the northwest of Egypt (31${}^{\circ }$20${}^{\prime }$ N, 27${}^{\circ }$13${}^{\prime }$ E) were used in this study. Thirty-four individuals belong to a governmental farm of the Camel Studies and Production Development Center in Mersa Matrouh Governorate, Animal Production Research Institute (APRI), Agricultural Research Center, Egypt. A total of 19 and 15 individuals belong to two private farms located in Mersa Matrouh Governorate, Egypt. Animals were identified by ear tags and aged between 6 to 16 years. The samples were taken randomly from animals with the least relationship in order to decrease the genetic similarity between the genotyped animals. Dromedaries were hand-milked twice a day. Concentrated feed mixture (CFM) was offered each morning for camels. CFM was composed of 25 % wheat bran, 20 % barley, 25 % yellow corn, 15 % rice bran, 9 % decorticated cotton seed meal, 2 % premix, 3 % molasses, and 1 % common salt. Forage of rice straw was offered to the animals in the afternoon. In addition to CFM, a little amount of Egyptian clover (*Trifolium alexandrinum*) was offered as a supplement from December to March.

### 2.3 Collection of milk samples and laboratory analysis

Milk samples were collected from 68 healthy individual camels belonging to the three studied farms at monthly intervals during the lactation period. Approximately 50 cm${}^{3}$ of milk samples was taken during morning milking for the determination of chemical composition. All samples were labeled, stored in an ice box, and transferred to the laboratory for immediate analysis. The chemical composition (fat, protein, lactose, total solids “TS”, and solids-not-fat “SNF” concentrations) of raw camel milk was determined with an automatic milk analyzer device (MilkoScan FT 120; Foss A/S, Hillerød, Denmark). The equipment has been validated against reference methods by the manufacturer and has also been calibrated for raw camel milk (Nagy et al., 2013). The pH values were evaluated by using a pH meter (Microprocessor pH Meter, pH211, Portugal).

### 2.4 Temperature–humidity index (THI)

The ambient temperature and relative humidity in the three investigated camel farms were recorded monthly by the Mersa Matrouh meteorological station located approximately 10–15 km from these farms, as shown in Table 1. The THI was calculated according to the equation of Kendall and Webster (2009) as given below:

$$\begin{array}{rl} \mathrm{THI} & =(1.8\times \mathrm{AT}+32)-[(0.55-0.0055\times \mathrm{RH})\\ & \times (1.8\times \mathrm{AT}-26)], \end{array}$$

where RH is the relative humidity and AT is the air temperature (${}^{\circ }$C).

**Table 1:** Monthly average temperature in 2019, relative humidity, and temperature–humidity index (THI) at the Mersa Matrouh meteorological station.

Month	Temperature	Relative	THI	THI class
	(${}^{\circ }$C)	humidity (%)		
December	20	7	62.885	Low
January	18	67	63.2384	
February	18	68	63.2736	
March	20	71	66.405	
April	23	68	70.6896	Medium
May	25	67	73.5515	
June	28	74	78.9108	
September	29	67	79.4447	
October	27	64	76.1252	
November	23	69	70.7743	
July	30	71	81.534	High
August	30	72	81.688	

### 2.5 DNA extraction and genotyping

Genomic DNA from 68 hair samples was extracted using a Gene JET genomic DNA purification kit, following the manufacturer's protocol (Fermentas, Waltham, MA, USA). DNA fragments of 659 bp spanning from -428 bp of 5${}^{\prime }$ flanking region to +231 bp of the camel CSN2 gene were amplified to detect the 2126${}_{A/G}$ SNP by using the previously published primers (Pauciullo et al., 2014). Each PCR mix consisted of 1.0 µM right and left primers, 0.2 mM dNTPs, and 1.25 U of Taq polymerase. The reaction mix was added to PCR tubes containing 50 ng of camel DNA. Reactions were performed using the following cycling conditions: initial incubation at 95 ${}^{\circ }$C for 5 min, followed by amplification for 35 cycles of 95 ${}^{\circ }$C for 60 s, 59.5 ${}^{\circ }$C for 45 s, 72 ${}^{\circ }$C for 90 s, and a final extension at 72 ${}^{\circ }$C for 10 min. Amplification was verified by electrophoresis on 2 % agarose gels stained with ethidium bromide (Gibco-BRL, Waltham, MA, USA) alongside a Gene-Ruler™ 50 bp ladder (Thermo Fisher Scientific, Waltham, MA, USA) and visualized on a UV trans-illuminator.

To detect 2126${}_{A/G}$ SNP of CSN2 gene in different individuals, the amplicons were digested using a restriction enzyme: *HphI* endonuclease (Fermentas, Waltham, MA, USA) by incubation for 15 min at 37 ${}^{\circ }$C. Restriction digestion was carried out in a total volume of 40 µL consisting of 20 µL of PCR product, 14 µL of dH${}_{2}$O, 5 µL of 10X G buffer, and 1 µL (10 U µL${}^{-1}$) of restriction enzyme. The restriction fragments were subjected to electrophoresis in 3.5 % agarose gel stained with ethidium bromide and visualized under UV trans-illuminator.

### 2.6 Statistical analysis

The association of different CSN2 genotypes with chemical composition of camel milk was determined using analysis of variance with genotype, parity, lactation stage, interaction between parity and lactation stage, THI, and the source population as the fixed effects in a general linear model (GLM) using the SAS software v8.2 statistical package (SAS Institute Inc., Cary, NC, USA) (SAS Institute, 1999).

The following linear model was used for the studied traits:

$$\begin{array}{rl} Y_{ijklm} & =\mu +G_{i}+{\mathrm{Pa}}_{j}+{\mathrm{Ls}}_{k}+\mathrm{Pa}(\mathrm{Ls}{)}_{jk}+T_{L}\\ & +F_{m}+e_{ijklm}, \end{array}$$

where $Y_{ijklm}$ is the milk chemical composition measurement, μ is the overall mean, $G_{i}$ is the fixed effect of the ith genotypes (three genotypes: A/A, A/G and G/G), Pa${}_{j}$ is the fixed effect of the jth parity (seven parities: first through seventh), Ls${}_{k}$ is the fixed effect of the kth lactation stage (three stages: early <3 months, middle = 3–6 months, and late >6 months), Pa (Ls)${}_{jk}$ is the interaction between parity and lactation stage, $T_{L}$ is the fixed effect of THI (three levels: low <70, moderate = 70–80, high >80), and $F_{m}$ is the fixed effect of population (three farms: first, second, and third), and $e_{ijklm}$ is the random error assumed to be normally distributed with a mean = 0 and a variance = $\sigma^{2}$e.

## 3 Results

### 3.1 Analysis of the polymorphism in CSN2 gene at 2126${}_{A/G}$ SNP among Egyptian Maghrebi camels

**Figure 1:**
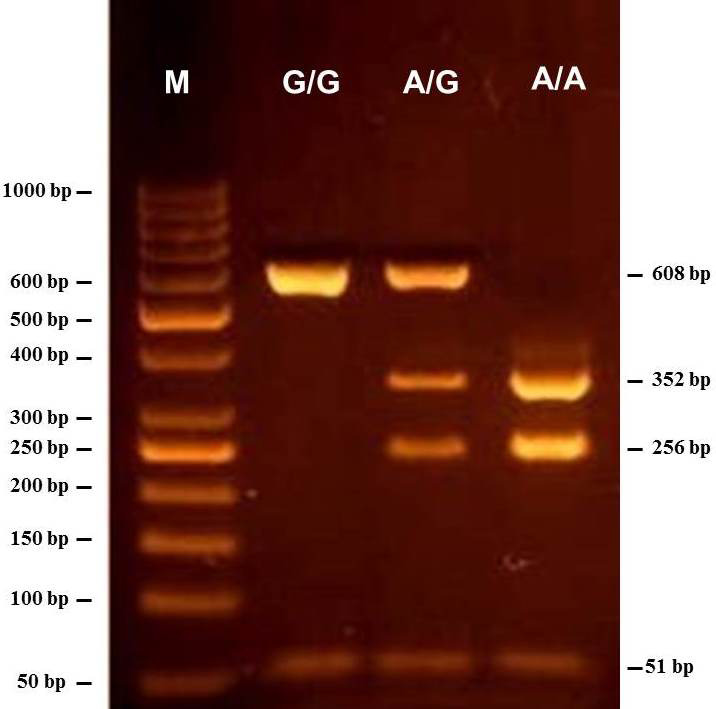
Gel electrophoresis showing the PCR-RFLP products of the SNP identified in the β-casein (CSN2) gene of *Camelus dromedarius*. The genotypes are indicated at the top of each lane. M is 50 bp DNA ladder marker.

The results of the PCR-RFLP analysis showed the presence of the three different restriction patterns in the investigated individuals (Fig. 1). The digestion of the 659 bp PCR product by *HphI* enzyme resulted in two fragments of 608 and 51 bp for the G/G samples, whereas the 608 bp fragment is further restricted into two fragments of 352 and 256 bp in case of A/A samples. The restriction pattern of the A/G heterozygous samples showed four fragments (608, 352, 256, and 51 bp). Among the 68 tested Maghrebi camels, the A/G genotype showed the highest frequency of 50 %, while the A/A and G/G genotypes occurred at a frequency of 38 % and 12 %, respectively (Table 2).

**Table 2:** Effect of *CSN2* genotypes on milk chemical compositions (%) in Maghrebi camels.

Genotype	N	Acidity (PH)	Fat	Protein	Lactose	Total solids	Solid-not-fat
		LSM ± SE	LSM ± SE	LSM ± SE	LSM ± SE	LSM ± SE	LSM ± SE
1 = AA	26 (38 %)	6.642 ± 0.03${}^{b}$	3.683 ± 0.096	3.568 ± 0.08${}^{a}$	4.939 ± 0.08	12.816 ± 0.17	8.760 ± 0.12
2 = AG	34 (50 %)	6.760 ± 0.02${}^{a}$	3.640 ± 0.07	3.029 ± 0.06${}^{b}$	5.171 ± 0.06	13.088 ± 0.13	9.023 ± 0.09
3 = GG	8 (12 %)	6.717 ± 0.05${}^{\mathrm{ab}}$	3.483 ± 0.15	3.124 ± 0.13${}^{b}$	5.027 ± 0.12	12.769 ± 0.27	8.989 ± 0.19
P value	68 (100 %)	0.008	0.980	0.003	0.180	0.170	0.140

N – number of observed genotypes, LSM – least-squares mean, SE – standard error.

### 3.2 Effect of genotype at CSN2 gene 2126${}_{A/G}$ SNP on milk composition in Maghrebi camels

Results of the association between *CSN2* genotype and milk chemical composition (acidity, fat, protein, lactose, TS, and SNF) in the Maghrebi camels are presented in Table 2. The results show that the 2126${}_{A/G}$ SNP had a significant effect on acidity and protein percentage with higher values observed in individual camels carrying the A/A genotypes, as shown in Table 2.

### 3.3 Effect of parity, lactation stage, and THI on milk composition of Maghrebi camels

Results for the effect of parity on milk composition in the Maghrebi camels are presented in Table 3. Protein percentage and PH value increased significantly from the first till the fourth parity and then decreased. Data regarding the variation in milk composition according to lactation stage for Maghrebi camels are presented in Table 4. Fat and TS percentages were significantly higher in late stage of lactation. Lactose percentage decreased significantly from early till late stages of lactation. THI showed a significant effect on fat and protein percentages of camels' milk with the highest values recorded for the low THI class (Table 5). The interaction between parity and lactation stage showed a significant effect on acidity, fat, protein, and lactose percentages. The highest and significant values for acidity and lactose were recorded at the third lactation stage of the second parity and at the first lactation stage of the first parity, respectively. The results of this study showed that the highest and significant percentages of protein and fat were recorded at the third lactation stage of the fourth and fifth parities, respectively, as shown in Table S1 in the Supplement.

**Table 3:** Effect of parity on milk chemical compositions (%) in Maghrebi camels.

Parity	N	Acidity (PH)	Fat	Protein	Lactose	Total solids	Solid-not-fat
		LSM ± SE	LSM ± SE	LSM ± SE	LSM ± SE	LSM ± SE	LSM ± SE
1	10	6.666 ± 0.04${}^{b}$	3.588 ± 0.13	3.259 ± 0.10${}^{\mathrm{bc}}$	4.946 ± 0.10	12.708 ± 0.22	8.895 ± 0.15
2	10	6.698 ± 0.04${}^{\mathrm{ab}}$	3.503 ± 0.14	3.246 ± 0.12${}^{b}$	5.188 ± 0.11	13.370 ± 0.25	9.201 ± 0.17
3	12	6.711 ± 0.04${}^{\mathrm{ab}}$	3.595 ± 0.13	3.411 ± 0.11${}^{\mathrm{ab}}$	5.040 ± 0.11	12.872 ± 0.24	8.778 ± 0.17
4	14	6.813 ± 0.04${}^{a}$	3.591 ± 0.13	3.599 ± 0.10${}^{a}$	4.998 ± 0.10	13.030 ± 0.22	8.908 ± 0.16
5	8	6.713 ± 0.05${}^{\mathrm{ab}}$	4.082 ± 0.15	3.260 ± 0.12${}^{\mathrm{bc}}$	5.185 ± 0.12	13.252 ± 0.27	9.006 ± 0.19
6	8	6.699 ± 0.05${}^{\mathrm{ab}}$	3.701 ± 0.15	3.058 ± 0.12${}^{c}$	5.127 ± 0.12	12.717 ± 0.27	8.960 ± 0.19
7	6	6.706 ± 0.05${}^{\mathrm{ab}}$	3.470 ± 0.17	2.539 ± 0.14${}^{d}$	4.931 ± 0.14	12.416 ± 0.30	8.523 ± 0.21
P value	68	0.013	0.087	0.002	0.601	0.131	0.244

N – number of parities, LSM – least-squares mean, SE – standard error.

**Table 4:** Effect of lactation stage on milk chemical compositions (%) in Maghrebi camels.

Lactation stage	N	Acidity (PH)	Fat	Protein	Lactose	Total solids	Solid-not-fat
		LSM ± SE	LSM ± SE	LSM ± SE	LSM ± SE	LSM ± SE	LSM ± SE
Early	68	6.744 ± 0.03	3.462 ± 0.09${}^{b}$	3.298 ± 0.07	5.282 ± 0.07${}^{a}$	12.835 ± 0.16${}^{b}$	9.090 ± 0.11
Middle	68	6.721 ± 0.03	3.703 ± 0.09${}^{a}$	3.129 ± 0.07	5.050 ± 0.07${}^{b}$	12.584 ± 0.16${}^{b}$	8.767 ± 0.11
Late	58	6.702 ± 0.03	3.755 ± 0.09${}^{a}$	3.391 ± 0.07	4.820 ± 0.07${}^{c}$	13.376 ± 0.16${}^{a}$	8.855 ± 0.11
P value		0.741	0.011	0.142	<0.001	0.0001	0.084

N – number of lactation stages, LSM – least-squares mean, SE – standard error.

**Table 5:** Effect of THI on milk chemical compositions (%) in Maghrebi camels.

THI class	N	Acidity (PH)	Fat	Protein	Lactose	Total solids	Solid-not-fat
		LSM ± SE	LSM ± SE	LSM ± SE	LSM ± SE	LSM ± SE	LSM ± SE
Low	68	6.724 ± 0.03	3.813 ± 0.09${}^{a}$	3.397 ± 0.07${}^{a}$	5.015 ± 0.07	12.844 ± 0.16	8.902 ± 0.11
Medium	66	6.733 ± 0.03	3.540 ± 0.09${}^{b}$	3.220 ± 0.07${}^{\mathrm{ab}}$	5.110 ± 0.07	12.988 ± 0.16	8.903 ± 0.11
High	60	6.706 ± 0.03	3.475 ± 0.10${}^{b}$	3.134 ± 0.09${}^{b}$	5.041 ± 0.08	13.001 ± 0.18	8.917 ± 0.13
P value		0.582	0.011	0.020	0.319	0.740	0.980

N – number of THIs, LSM – least-squares mean, SE – standard error.

## 4 Discussion

The production of high-quality camel milk is an essential step towards success in the milk and processed milk products industries (Nagy et al., 2017). This study aimed to identify *CSN2* gene polymorphism in Egyptian Maghrebi camels and to determine their association with the chemical composition of milk. In this study the heterozygous A/G genotype recorded the highest frequency of 50 % while the A/A and G/G genotypes occurred at frequencies of 38 % and 12 %, respectively. Similar trends were reported by Pauciullo et al. (2014), who identified three *CSN2* genotypes – A/G (51 %), A/A (40 %), and G/G (9 %) – across four populations of Sudanese camels (Shanbali, Kahli, Lahaoi, and Arabi) using PCR-RFLP method. Our results showed that Maghrebi camels with A/A genotypes had a higher protein percentage with higher acidity. Previous studies have reported a significant association of *CSN2* gene polymorphisms with higher protein and casein content in cattle (Nilsen et al., 2009; Bugeac et al., 2015; Bhat et al., 2017; Viale et al., 2017; Soyudal et al., 2018). The association between the *CSN2* gene polymorphisms and milk protein percentage of our study might be attributed to the modification of the mRNA stability and transcription rates (Szymanowska et al., 2004; Kuss et al., 2005). Moreover, the 2126${}_{A/G}$ SNP of CSN2 gene is located three nucleotides downstream of the TATA box and that might modify the binding affinity of RNA polymerase and change the gene expression (Lee et al., 2012). Additionally, the association might be attributed to the linkage disequilibrium condition between variants of coding regions and 2126${}_{A/G}$ SNP located in the regulatory regions. Therefore, further investigation is required to verify the influence of the identified 2126${}_{A/G}$ SNP on β-casein gene regulation.

The results of this study showed that Maghrebi camels produced milk with higher protein and PH values at the fourth parity. Similar results were obtained by Zeleke (2007) and Berhane (2016), who reported the highest protein content at the third parity, and Babiker and El-Zubeir (2014), who reported the highest content at the fifth one. By contrast, Bakheit et al. (2008) and Aljumaah et al. (2012) reported the highest protein at the first parity. Moreover, Ahmad et al. (2012) and Al-Sultan and Mohammed (2007) reported a non-significant effect of parity on protein content and acidity of the camel milk. The increasing of protein percentage of our study till the fourth parity and then decreasing may be attributed to camels in the earlier parities being still in the growing stage and the supplied nutrients are partitioned for body building and milk production. Similarly, older camels as compared to intermediate ages may suffer from a reduction in the number and efficiency of milk secreting cells and also wearing of teeth that may affect the chemical composition of camel milk (Zeleke, 2007).

Fat and TS contents in Maghrebi camels were significantly higher at the third stage of lactation, probably due to decreased milk yield at this stage. Our results were in agreement with those reported by Mestawet et al. (2012), who reported higher fat and TS at the late stage of lactation. On the contrary, Zeleke (2007), Aljumaah et al. (2012) and Babiker and El-Zubeir (2014) reported the highest fat content during the first three months of lactation. Moreover, Ahmad et al. (2012) recorded a non-significant effect of lactation stage on fat and TS contents among pastoral herders grazing on natural vegetation. The highest and significant percentages of protein and fat of this study were recorded at the third lactation stage of the fourth and fifth parities, respectively.

In agreement with previous studies, lactose content of our study significantly decreased from early to late lactation stages (Ahmad et al., 2012; Aljumaah et al., 2012; Babiker and El-Zubeir, 2014; Nagy et al., 2017). The low THI class (December to March) of our study recorded the highest and significant fat and protein percentages, while the high THI class (July–August) recorded the lowest values. The explanation of this finding might be attributed partly to dietary changes such as supplementation of green forage during the winter months and partly to the seasonal changes in the environmental factors such as ambient temperature and photoperiod because dromedaries are seasonal breeders (Dahl et al., 2012; Allali et al., 2013). Similar results were obtained by Nagy et al. (2017), who reported higher values of fat and protein during the winter months, while the lowest ones were during the summer months. By contrast, Bakheit et al. (2008) recorded the opposite trend, where the summer months showed the highest fat and protein content.

There is a relationship between the chemical composition of raw camel milk and the processing characteristics, such as clotting ability during cheese manufacturing. The rheological properties of milk curd are greatly dependent on the amount of total solids in the camel milk and are enhanced as total solids are increased. Among the total solids, proteins, especially casein, are the most important components and play a major role in the formation of the micelle network; the higher it is, the stronger the formation of the casein matrix (Yagil and Etzion, 1980; Yagil, 1994; Babiker and El-Zubeir, 2014). Moreover, Lambert (1988), del-Castillo (1990), and Fox et al. (2004) reported that the composition and processing of cheese depends mainly on the percentage of total solids and fat in camel milk, where fat globules are caught in the casein matrix, decreasing the clot rigidity. The optimal activity of milk-clotting enzymes generally occurs in acidic medium where the optimal pH is approximately 5.5 (Ramet, 2001). From the literature, it appears that increasing the milk acidity results in decreasing the clotting time during cheese processing (Farah, 1993; Jardali, 1994).

Therefore, the factors influencing the chemical composition of camel milk might be considered for the nutritional and processing properties of milk and milk products. The higher values for acidity and protein percentage from the milk of the individuals carrying the A/A genotype may encourage the dairy plants for cheese processing and manufacturing from these individuals. Moreover, the higher percentages of protein and fat at the third lactation stage of the fourth and fifth parities may encourage the processing of camel milk into cheese and butter products respectively at these periods.

In conclusion, this study showed that *CSN2* gene, parity, lactation stage, and THI recorded a significant effect on the chemical composition of camel milk. Maghrebi camels produced milk with higher percentages of protein and fat at the end of lactation stages of the middle parities of their productive life. Accordingly, these data may suggest the processing of their milk into cheese and butter in these periods. Our results suggest that the 2126${}_{A/G}$ SNP of the CSN2 gene significantly affected milk composition in Maghrebi camels. Such a polymorphic locus may be useful as a marker in assisted selection programs for the improvement of camel milk composition. To fully explore and utilize the changes in the chemical and processing properties of camel milk due to the effect of *CSN2* gene, parity, lactation stage, and THI, further studies are eagerly anticipated.

## Supplement

10.5194/aab-63-493-2020-supplement The supplement related to this article is available online at: https://doi.org/10.5194/aab-63-493-2020-supplement.

## Data Availability

The underlying research data can be obtained from the corresponding author, who can be reached by email: sherif.ramadan@fvtm.bu.edu.eg and amira.nowier@ARC.sci.g.
