# Supplementary material for: Association of β-casein gene polymorphism with milk composition traits of Egyptian Maghrebi camels (Camelus dromedarius)
Source: Arch Anim Breed. 2020 Dec 22;63(2):493–500. doi: 10.5194/aab-63-493-2020 (PMC7810228; doi:10.5194/aab-63-493-2020)
Supplement: The supplement related to this article is available online at: https://doi.org/10.5194/aab-63-493-2020-supplement. [file aab-63-493-supplement.pdf]

Supplement of Arch. Anim. Breed., 63, 493–500, 2020
https://doi.org/10.5194/aab-63-493-2020-supplement
© Author(s) 2020. This work is distributed under
the Creative Commons Attribution 4.0 License.

Open Access
Archives Animal Breeding

Supplement of

Association of β-casein gene polymorphism with milk composition traits of Egyptian Maghrebi camels (*Camelus dromedarius*)

Amira M. Nowier and Sherif I. Ramadan

Correspondence to: Sherif I. Ramadan (sherif.ramadan@fvtm.bu.edu.eg)

The copyright of individual parts of the supplement might differ from the CC BY 4.0 License.

S1 Table Effect of the interaction between the parity nd lactation stages on milk chemical compositions (%) in Maghrebi camels

Parity	Lactation stage	N	PH			Fat			Protein			lactose			TS			SNF		
			LSM	±	SE	LSM	±	SE	LSM	±	SE	LSM	±	SE	LSM	±	SE	LSM	±	SE
1	1	30	6.764	±	0.079	3.062	±	0.750	3.451	±	0.403	5.662	±	0.382	12.583	±	1.446	9.726	±	0.739
1	2	30	6.695	±	0.165	3.519	±	0.921	3.037	±	0.937	4.957	±	0.690	12.475	±	1.809	8.555	±	1.015
1	3	36	6.562	±	0.278	4.084	±	0.707	3.284	±	0.580	4.339	±	0.843	13.006	±	1.001	8.487	±	0.748
2	1	30	6.733	±	0.143	3.409	±	0.428	3.116	±	0.829	5.259	±	0.659	13.133	±	1.585	9.191	±	1.043
2	2	28	6.790	±	0.128	3.401	±	1.084	3.162	±	0.583	5.089	±	0.495	13.195	±	1.312	9.009	±	0.683
2	3	20	6.518	±	0.598	3.785	±	0.746	3.559	±	0.238	5.218	±	0.543	13.971	±	1.325	9.486	±	0.545
3	1	36	6.856	±	0.325	3.385	±	0.472	3.506	±	0.565	5.263	±	0.516	12.963	±	1.566	9.031	±	1.024
3	2	34	6.690	±	0.100	3.665	±	0.820	3.245	±	1.122	5.119	±	0.727	12.473	±	1.161	8.859	±	1.002
3	3	38	6.592	±	0.170	3.731	±	0.851	3.470	±	0.276	4.759	±	0.480	13.144	±	1.359	8.466	±	1.409
4	1	42	6.726	±	0.114	3.466	±	0.672	3.487	±	0.693	5.169	±	0.861	12.761	±	1.599	8.912	±	1.303
4	2	42	6.698	±	0.127	3.873	±	0.898	3.600	±	0.962	4.901	±	0.474	12.444	±	0.854	8.642	±	0.717
4	3	44	7.007	±	0.499	3.441	±	0.717	3.704	±	0.738	4.926	±	0.954	13.846	±	2.079	9.156	±	1.077
5	1	24	6.694	±	0.080	4.018	±	0.640	3.184	±	0.426	5.407	±	0.451	13.247	±	1.541	9.223	±	0.938
5	2	22	6.758	±	0.144	4.103	±	0.794	3.323	±	0.833	5.090	±	0.523	12.900	±	1.593	8.767	±	0.982
5	3	22	6.687	±	0.207	4.132	±	0.879	3.279	±	0.329	5.038	±	0.821	13.609	±	1.288	9.008	±	0.990
6	1	24	6.703	±	0.082	3.604	±	0.452	3.179	±	0.452	5.154	±	0.385	12.806	±	1.311	8.951	±	0.737
6	2	24	6.709	±	0.079	3.908	±	1.463	2.739	±	0.545	5.353	±	0.593	12.692	±	1.548	9.116	±	0.860
6	3	24	6.687	±	0.096	3.590	±	0.613	3.254	±	0.332	4.876	±	0.254	12.653	±	0.900	8.813	±	0.450
7	1	18	6.667	±	0.134	3.432	±	0.471	2.800	±	0.699	4.992	±	0.768	12.166	±	1.223	8.401	±	1.528
7	2	18	6.740	±	0.138	3.362	±	1.489	2.193	±	0.822	4.909	±	0.421	11.826	±	1.121	8.397	±	0.793
7	3	14	6.713	±	0.082	3.657	±	0.568	2.649	±	0.933	4.880	±	0.581	13.499	±	1.302	8.841	±	0.735
P value			0.002			0.003			0.017			0.004			0.227			0.0512		

N = Number of records of the interaction, LSM = Least-square mean, SE= Standard error
